# Editorial: Pattern recognition for healthcare analytics

**DOI:** 10.3389/fdgth.2023.1186713

**Published:** 2023-03-30

**Authors:** İnci M. Baytaş, Yifan Peng, Arzucan Özgür

**Affiliations:** ^1^ Department of Computer Engineering, Faculty of Engineering, Boğaziçi University, Bebek, Istanbul, Türkiye; ^2^ Department of Population Health Sciences, Weill Cornell Medicine, New York, NY, United States

**Keywords:** representation learning (RL), machine learning - ML, pattern recognition (ANN), natural language processing (computer science), healthcare analytics

**Editorial on the Research Topic** https://www.frontiersin.org/research-topics/28402/pattern-recognition-for-healthcare-analytics

Analyzing a vast amount of digital patient data is vital to infer the characteristics of a patient cohort. Pattern recognition offers essential tools for healthcare analytics tasks. In particular, machine learning and deep learning techniques have been successfully applied to various healthcare tasks, such as risk prediction, disease progression prediction, and patient subtyping. However, the digital patient data's heterogeneous, high-dimensional, non-linear, temporal, and distributed nature poses additional challenges when using pattern recognition techniques. Such challenges inspire researchers to design novel artificial learning methods to solve specific challenges in healthcare. This Research Topic aims to showcase some of the latest developments in pattern recognition for healthcare analytics, including ideas on predictive modeling, clustering, feature extraction, temporal analysis, data visualization, and interpretability for patient data in tabular, text, and image formats. In this regard, we present four studies introducing several contributions to pattern recognition in the healthcare domain.

Electronic Health Records (EHRs) systems are widely employed at hospitals to register patient information in various data types, including text. Since patient data is vulnerable, the privacy of the information in EHRs should be protected. Therefore, de-identification techniques can be applied before circulating EHR data to ensure patient privacy. Paul et al*.* explored this problem in their study titled *“Investigation of the Utility of Features in a Clinical De-identification Model: A Demonstration Using EHR Pathology Reports for Advanced NSCLC Patients”*. The authors utilized open-source Natural Language Processing (NLP) toolkits to explore various text representation techniques to extract features from EHR's text data. The best features based on the experiments and their combinations were used to train a Named Entity Recognition (NER) model, which enables identifying clinical entities of interest in the text. The authors suggested n-gram, prefix-suffix, word embeddings, and word shape as the best-performing features for improving recall in the NER task.

EHRs are priceless resources for studying contributing factors of various conditions. For instance, Mahabadi et al*.* investigated the impact of the physical characteristics of the urban environment on Severe Mental Illnesses (SMI) using EHR data in their study titled *“Evaluating Physical Urban Features in Several Mental Illnesses using Electronic Health Record Data”*. The authors considered 28 urban and 6 clinical features from a cohort of 30,210 patients. The scale of the patient cohort makes it impossible for a clinician to draw inferences manually from the data of more than 30 K patients. However, the authors addressed this challenge by benefiting the LASSO regression's interpretability property to obtain the most significant features. The authors also suggested employing the Self-Organising Map technique to interpret the results visually.

Predicting disease progression is vital for neurodegenerative diseases, such as Alzheimer's disease (AD). Hason and Krishnan focused on the early diagnosis and progression of AD based on speech signals in their study titled *“Spontaneous Speech Feature Analysis for Alzheimer's Disease Screening using a Random Forest Classifier”*. Depending on the healthcare task, specific requirements for feature extraction emerge. This study aimed to discriminate AD patients from cognitively normal patients using acoustic input. The authors suggested exploring the non-stationarity and non-linearity properties of the audio features to determine the most significant audio features to run a random forest classifier to detect AD.

It has been evident in the studies mentioned earlier that representing patients using salient and informative features is crucial to train successful machine learning models for healthcare tasks. Hernandez et al*.* studied this challenge in *“Learning Meaningful Latent Space Representations for Patient Risk Stratification: Model Development and Validation for Dengue and Other Acute Febrile Illness”* by learning to project patient data onto a new latent space. Autoencoders were trained to produce the lower dimensional latent space where the new representations were clinically plausible. The authors further discussed that learning clinically meaningful and informative patient representations can be a component of electronic clinical decision support systems.

In summary, this Research Topic presents various data-driven approaches to solving different healthcare tasks with diverse types of patient data. All the studies emphasize that determining significant features per the specific task at hand improves the performance of the data-driven models.

